# Evolution and expression of *LEAFY* genes in ferns and lycophytes

**DOI:** 10.1186/s13227-021-00188-9

**Published:** 2022-01-08

**Authors:** Carolina Rodríguez-Pelayo, Barbara A. Ambrose, Alejandra Vasco, Juan F. Alzate, Natalia Pabón-Mora

**Affiliations:** 1 Facultad de Ciencias Exactas y Naturales, Instituto de Biología, Universidad de Antioquia, Medellín, Colombia; 2 The New York Botanical Garden, Bronx, NY USA; 3 Botanical Research Institute of Texas, Fort Worth, TX USA; 4 Centro Nacional de Secuenciación Genómica, Facultad de Medicina, Sede de Investigación Universitaria, Universidad de Antioquia, Medellín, Colombia

**Keywords:** *LEAFY*, Ferns, Lycophytes, Reproductive transition, Sporogenesis

## Abstract

**Background:**

The *LEAFY* (*LFY*) transcription factors are present in algae and across land plants. The available expression and functional data of these genes in embryophytes suggest that *LFY* genes control a plethora of processes including the first zygotic cell division in bryophytes, shoot cell divisions of the gametophyte and sporophyte in ferns, cone differentiation in gymnosperms and floral meristem identity in flowering plants. However, their putative plesiomorphic role in plant reproductive transition in vascular plants remains untested.

**Results:**

We perform Maximum Likelihood (ML) phylogenetic analyses for the *LFY* gene lineage in embryophytes with expanded sampling in lycophytes and ferns. We recover the previously identified seed plant duplication that results in *LEAFY* and *NEEDLY* paralogs. In addition, we recover multiple species-specific duplications in ferns and lycophytes and large-scale duplications possibly correlated with the occurrence of whole genome duplication (WGD) events in Equisetales and Salviniales. To test putative roles in diverse ferns and lycophytes we perform *LFY* expression analyses in *Adiantum raddianum*, *Equisetum giganteum* and *Selaginella moellendorffii.* Our results show that *LFY* genes are active in vegetative and reproductive tissues, with higher expression in early fertile developmental stages and during sporangia differentiation.

**Conclusions:**

Our data point to previously unrecognized roles of *LFY* genes in sporangia differentiation in lycophytes and ferns and suggests that functions linked to reproductive structure development are not exclusive to seed plant *LFY* homologs.

**Supplementary Information:**

The online version contains supplementary material available at 10.1186/s13227-021-00188-9.

## Background

The *LEAFY* (*LFY*) gene lineage is a plant specific transcription factor family [1]. *LFY* is best known for its role in the flowering plant model species *Arabidopsis thaliana* (Arabidopsis), where it is a key integrator for flowering transition, controlling both the floral meristem identity and the activation of the floral organ identity genes [1–4]. *LFY* genes are primarily retained as a single copy gene in algae and across land plants, with only a large-scale duplication occurring in seed plants resulting in two clades *NEEDLY* (*NDLY*) and *LFY*. Both copies have been retained in gymnosperms but *NDLY* was lost in angiosperms [5, 6]. In addition, some species-specific duplications have been identified in many lineages of land plants [5, 7].

*LFY* genes encode a ca. 220–350 amino acid protein that acts as a homodimer and it has two recognizable domains: the N-terminal (also known as LFY_SAM) and the C-terminal (also known as C_LFY_FLO) DNA binding domains [8]. The LFY_SAM domain is key for dimerization, DNA binding and DNA accessibility, while the C_LFY_FLO domain has been proposed to mediate DNA binding specificity that has evolved across land plants [5, 9]. Based on the dominant amino acids, three different motif types have been identified in the DNA binding domain in different streptophyte lineages: liverworts + tracheophytes have the type I motif, mosses have the type II motif, and algae possess the type III motif [5]. Finally, hornworts have been reported to have a promiscuous motif with versatile DNA binding capabilities binding all three types of DNA motifs [5].

Functional studies of *LFY* homologs in other flowering plants, such as *Antirrhinum majus* (snapdragon)*,* showed that *floricaula* (*flo*) mutants, do not transition from vegetative to reproductive stages. In these mutants, inflorescence meristems do not form floral meristems on their flanks, resulting in branched stems forming more leaves instead of producing flowers, similar to the Arabidopsis *lfy* mutants [1, 10–12]. Conversely, the overexpression of *LFY* homologs turns the indeterminate inflorescence into a determinate meristem forming solitary flowers [13]. In Arabidopsis, *LFY* is upregulated by flowering time genes including *AGAMOUS-LIKE 24 (AGL24), SHORT VEGETATIVE PHASE (SVP)* and *SUPRESSOR OF OVEREXPRESSION OF CONSTANS (SOC1)* [14–17]*.* Once LFY is active in the floral meristem, it stimulates cytokinin signaling and activates floral fate acquisition together with APETALA1 (AP1) (a MADS-box homolog) responsible for sepal and petal identity [18–22]. LFY can also activate other floral organ identity MADS-box genes including *SEPALLATA* (*SEP*), *PISTILLATA* (*PI*), *APETALA3* (*AP3*) and *AGAMOUS* (*AG*) [23].

Based on functional data from Arabidopsis and snapdragon, *LFY* has been characterized as a critical factor controlling floral meristem identity and floral fate. These roles are conserved in other angiosperms, such as apple, papaya and eucalyptus [24–26]. In addition, other roles have been reported for *LFY* members across angiosperms. For instance, *LFY* homologs have been recruited in the formation of compound leaves in legumes [27, 28], in shoot apical meristem (SAM) development in cucumber, impatiens, and tomato [29–32], in inflorescence branch patterning in rice [33], and in floral merism and spiral arrangement of floral parts in columbines [34].

Studies of gene homologs in gymnosperms show that both *LFY* and *NDLY* are expressed constitutively in reproductive and vegetative structures, and spatial–temporal expression differences between paralogs have been linked to male and female cone differentiation [35–38]. In addition, heterologous expression of gymnosperm *LFY* homologs can rescue the wild type phenotype from *lfy* mutants in Arabidopsis [37], which suggest functional conservation in the transition to reproduction across seed plants.

Expression and function of two *LFY* paralogs have also been studied in the fern *Ceratopteris richardii.* RT-PCR assays and in situ hybridization showed high expression of the two *C. richardii LFY* copies in the shoot tips and circinate fertile leaves, both characterized by active cell division [7, 39]. In addition, heterologous expression of fern *LFY* homologs does not fully recover wild type phenotypes in *lfy Arabidopsis* mutants [40]. Conversely, endogenous loss-of-function double *lfy* mutants in *C. richardii* exhibit defects in gametophyte development by cell division arrest of the apical cell, as well as truncate sporophyte development accompanied by abnormal leaf development [39, 40]. However, because *lfy* mutants result in interrupted shoot development, it is unclear if *LFY* homologs play roles during the reproductive transition in ferns.

Studies in lycophytes are restricted to *Isoetes,* where two *LFY* homologs were identified. The *Isoetes LFY* homologs are also expressed in the vegetative and reproductive tissues of the sporophyte with higher expression in juvenile tissues and fertile leaves bearing megasporangia and microsporangia [41]. Importantly, heterologous expression of *Isoetes LFY* genes in Arabidopsis did not rescue *lfy* mutant phenotypes [41]. Finally, the role of *LFY* homologs has also been evaluated in bryophytes, in the model *Physcomitrium patens*. This species has two paralogs, *PpLFY1* and *PpLFY2* regulate the first division of the zygote and in turn controls proper cell division of the sporophyte as seen in the double mutant with developmentally arrested zygotes [42].

The data available for land plant *LFY* homologs was summarized by Plackett et al*.* [39] who concluded that plesiomorphic roles of *LFY* genes include the control of cell divisions in the zygote to form the sporophyte in bryophytes, and that *LFY* homologs were subsequently co-opted to maintain indeterminate cell fate in both the gametophyte and the sporophyte in ferns. These authors also hypothesized that, only in seed plants, *LFY* genes were active during cone development in gymnosperms and controlled floral meristem identity in angiosperms. Interestingly, due to their early critical roles in shoot development it is still unclear if fern *LFY* homologs play any function in the formation of fertile leaves and their specialization for spore production in ferns. If so, the *LFY* gene function in reproductive transition and sporangia development is not exclusive to seed plants. In this context, we aim to reconstruct the evolution of the *LFY* gene lineage with ample sampling from lycophytes and ferns, and to assess expression patterns of *LFY* genes in lycophytes and ferns during their reproductive transition. We make use of public databases as well as our own transcriptomes from four fern species with different degrees of leaf dimorphism (i.e., morphological differences between fertile and sterile leaves): the monomorphic (i.e., sterile and fertile leaves with same morphology) *Adiantum raddianum*, the hemidimorphic (i.e., sterile and fertile leaves with slightly different morphologies having for instance differentiated fertile pinnae) *Anemia villosa*, and two holodimorphic (i.e., sterile and fertile leaves with different morphology) species, *Equisetum giganteum* and *E. bogotense*. The expression of *LFY* copies was assessed by RT-PCR in selected ferns with the extreme changes in leaf dimorphism (*Adiantum raddianum* and *Equisetum giganteum*), and by in situ hybridization (ISH) in the lycophyte *Selaginella moellendorffii.* Our results allow us to propose a putative role for lycophyte and fern *LFY* homologs in reproductive tissue formation (i.e., sporangia development), indicating that this function may have been present in the *LFY* gene lineage prior to the diversification of seed plants.

## Results

### LEAFY gene family evolution

We present here the most comprehensive phylogenetic analysis of *LFY* genes to date, consisting of 228 sequences that include 95 from Sayou et al. [5] and 133 sequences from an expanded sampling targeting ferns and lycophytes (Additional file 1: Fig. S1). The complete aligned data set consists of 1338 characters from which 833 are informative.

**Fig. 1:**
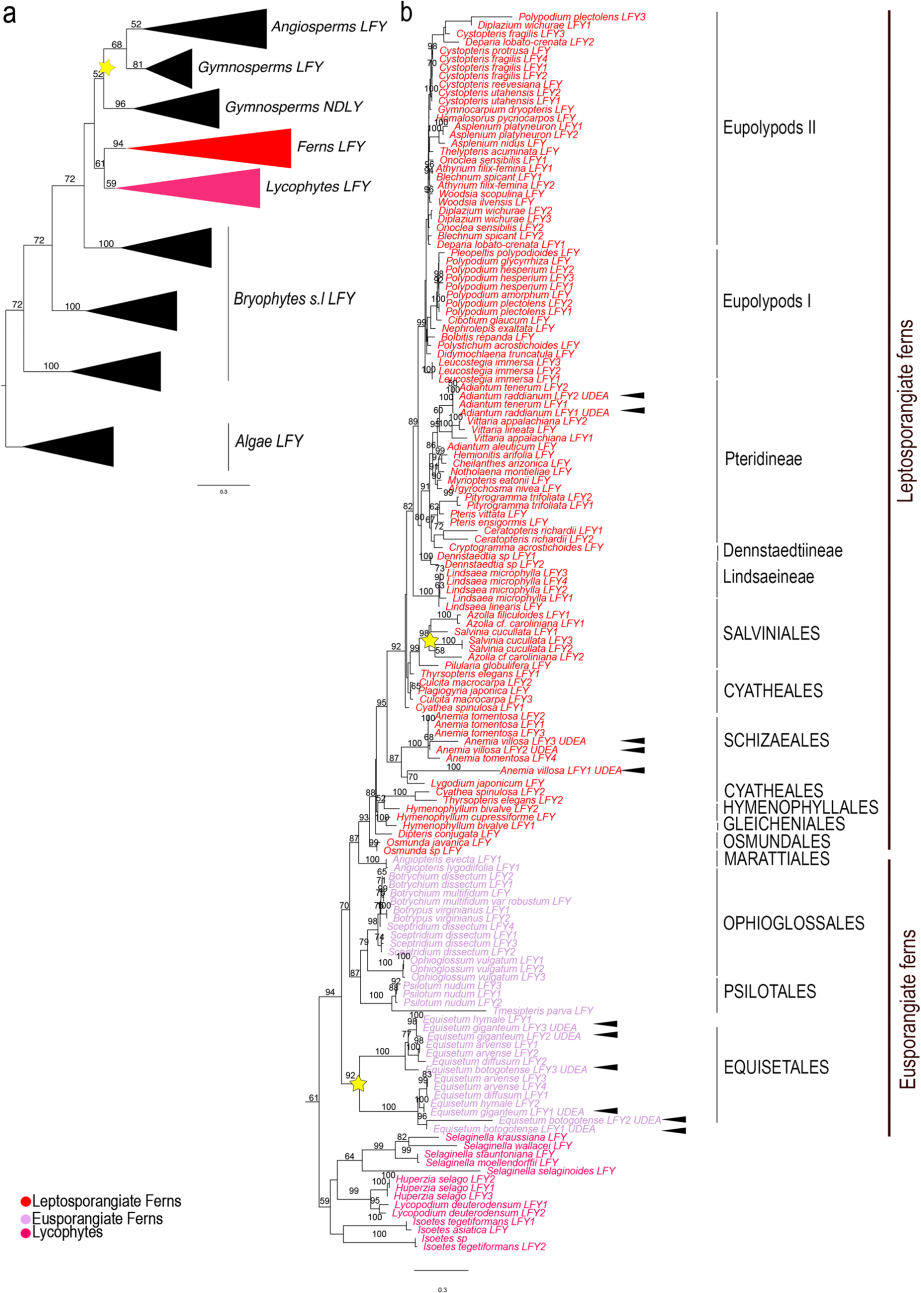
ML analysis of the *LFY* transcription factor family **a** Summary tree including sequences from algae, bryophytes and tracheophytes with Boostrap values (BS) as numbers on top of branches for major clades. **b** Detail of lycophyte and fern LFY clades. Yellow stars indicate large scale (major) duplication events. Number on each node indicate the BS. Black arrowheads point to sequences isolated in this study. The colors correspond to the conventions on the bottom left. Scale: 0.3

Similar to previous reports, our analysis recovered *LFY* genes predominantly as single copy genes in streptophyte algae and land plants, with a single exception in seed plants. In turn, the gene tree mostly recovers the phylogenetic relationships recorded for major plant lineages. The bryophyte *LFY* genes are sister to *LFY* homologs in vascular plants (Bootstrap Support, BS = 72) (Additional file 1: Fig. S1). However, within vascular plants, sequences from ferns and lycophytes are recovered as sister to each other (BS = 61) and the lycophyte + fern *LFY* clade is sister to the seed plant *LFY* homologs with low support (Fig. 1). This is inconsistent with the vascular plant phylogeny accepted to date, where lycophytes are sister to euphyllophytes (the clade formed by ferns and seed plants) [43]. In addition, our topology recovers the *LFY* and *NDLY* duplication event prior to the diversification of seed plants with a loss of *NDLY* homologs in angiosperms [5, 38, 40]. Moreover, we found multiple fern and lycophyte species-specific duplications some of which could explain the multiple homologs reported previously in the fern C*eratopteris richardii* and in several *Isoetes* spp. in the lycophytes [39, 41].

The evolution of *LFY* gene homologs within ferns and lycophytes, closely match the phylogeny of those lineages [43]. However, our analysis recovered large-scale duplications of *LFY* in Equisetales, in Salviniales, within Pteridineae in the Vittarioideae subfamily, and in Eupolypods II (sensu, [43]). However, the exact timing of the latter two duplications cannot be placed with certainty. Unlike their expected phylogenetic placements, our analyses recovered *Cibotium glaucum LFY* homolog nested within the Eupolypod I sequences and the *Polypodium plectolens* homolog within Eupolypod II sequences. In the species phylogenies these species belong to Cyatheales and Eupolypoids I, respectively [43].

Finally, our analysis reveals species-specific duplications in the lycophyte *Huperzia selago,* a well-known polyploid, and in a number of ferns, most of which have been identified as tetraploids or recent hybrids, including *Anemia villosa*, *Asplenium platyneuron*, *Botrypus virginianus*, *Ceratopteris richardii*, *Cystopteris fragilis, Dennstaedtia sp., Equisetum arvense*, *E. bogotense*, *Leucostegia immersa, Lindsaea microphylla*, *Ophioglossum vulgatum*, *Polypodium hesperium, P. plectolens, Psilotum nudum*, *Pityrogramma trifoliata* and *Sceptridium dissectum*.

### LFY protein domains and motifs

**Fig. 2:**
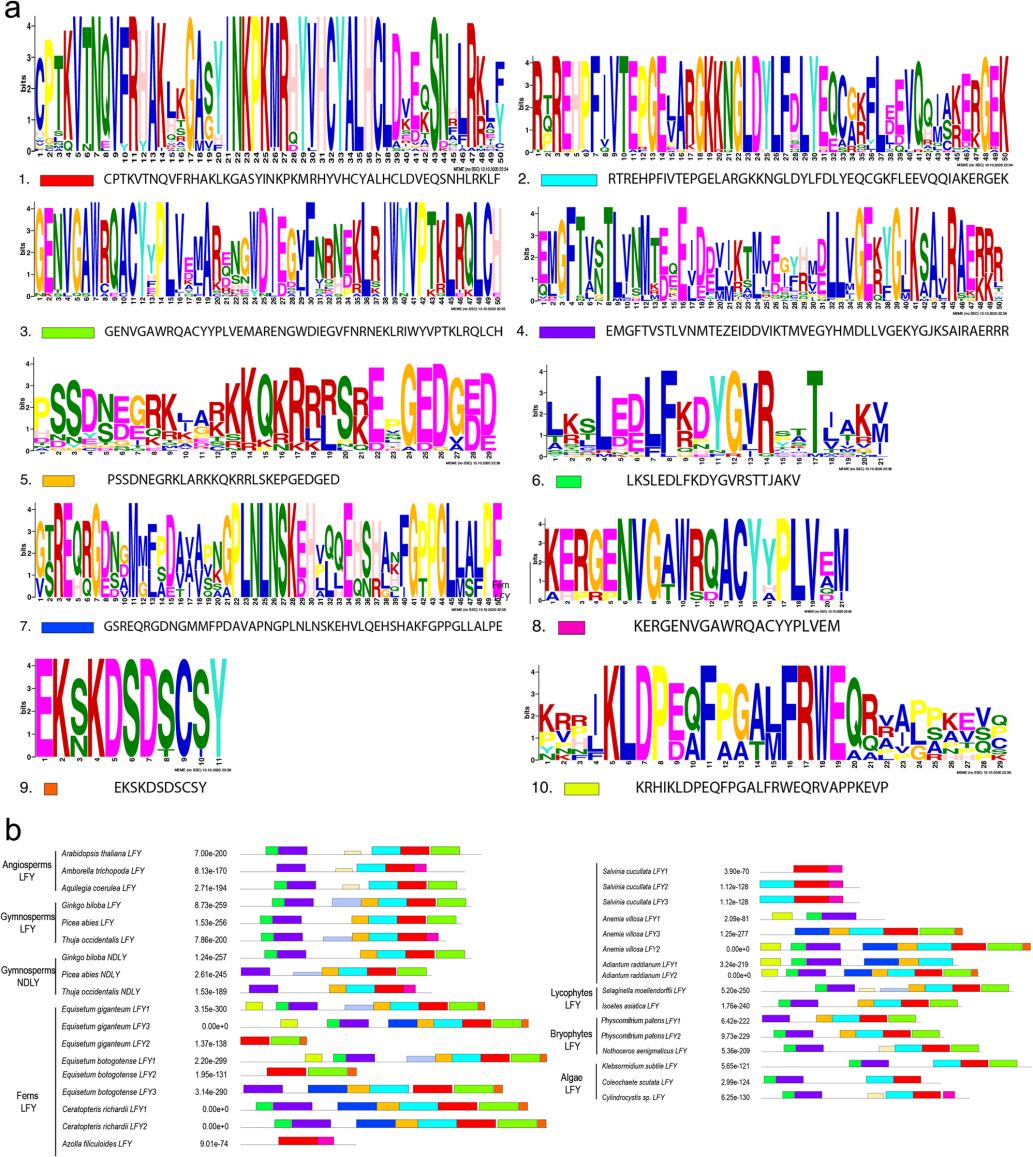
**a** Conserved motifs of LFY proteins identified by a MEME analysis, their motifs, numbers assigned and their respective sequence. **b** Map of the motif positions in land plant representative proteins

LFY proteins are characterized by two conserved domains [5, 6, 9], the LFY_SAM (in the N terminus), which in our analysis corresponds to motifs 1, 2 and 3 and the C_LFY_FLO DNA binding domain (in the C terminus) which in our analysis corresponds to motifs 4 and 6 [6] (Fig. 2). We do not find outstanding variation at the protein level between divergent plant lineages (i.e., lycophytes, ferns, gymnosperms and angiosperms), but we were able to identify motifs 9 and 10 as exclusive to fern protein sequences (Fig. 2).

**Fig. 3:**
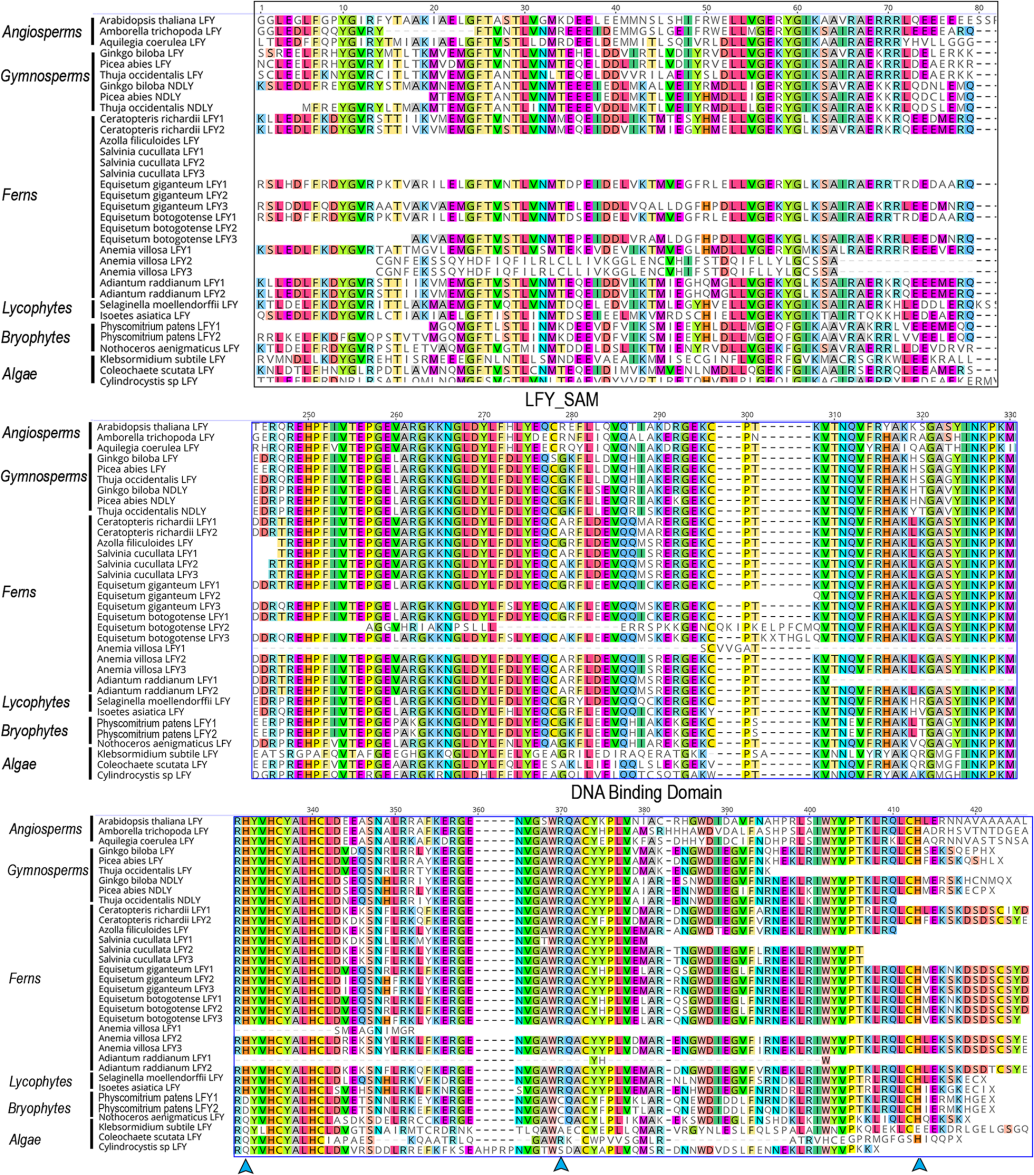
Detail of the LFY Protein domains alignment for 34 representative land plant sequences belonging to: *Arabidopsis thaliana*, *Ceratopteris richardii*, *Azolla filiculoides*, *Salvinia cucullata*, *Equisetum giganteum*, *E. bogotense*, *Adiantum raddianum*, *Anemia villosa*, *Selaginella moellendorffi*, *Physcomitrium patens* and several algae species. The two characteristic domains of LFY proteins reported by Sayou et al. [5, 9] are boxed. Blue arrowheads point to the key positions for DNA binding reported by Sayou et al. [5]

In addition, the protein alignment does allow the identification of some conserved amino acids in fern LFY proteins in the most variable region between the SAM_LFY and the C_LFY_FLO DNA binding domain (Additional file 2: Fig. S2). Finally, following the key amino acids positions for DNA binding, our analyses recovered the amino acids HRH, matching the type I described for tracheophytes [5]. They correspond to positions 332, 370 and 413 in our alignment (Fig. 3).

### RT-PCR expression of *LFY* homologs in selected ferns

**Fig. 4:**
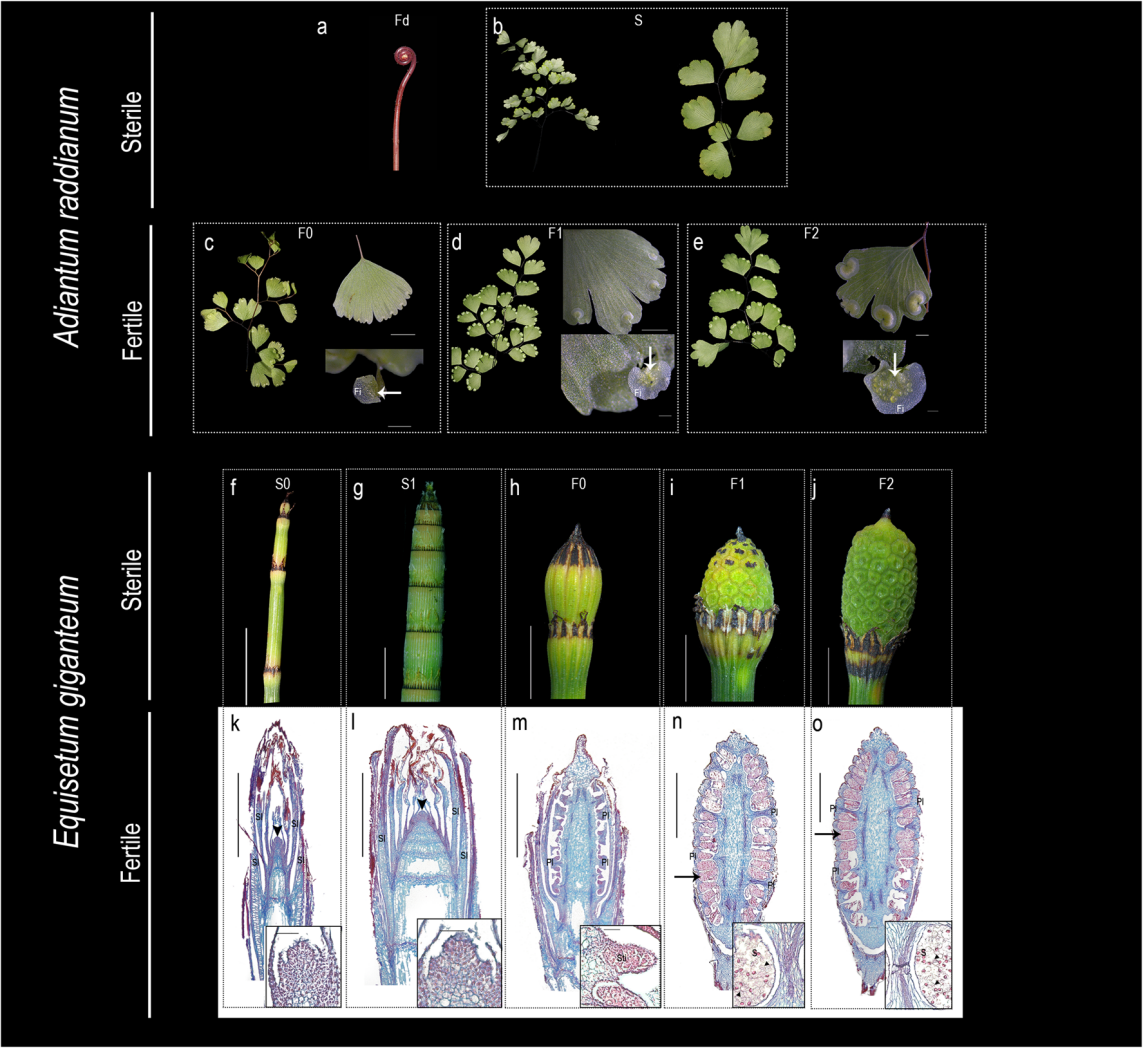
Developmental stages of the fern species *Adiantum raddianum* and *Equisetum giganteum*. (**a–e**) *A. raddianum* stages: Two sterile stages include Fd: fiddlehead and S: sterile expanded pinnae (**a**, **b**). Three fertile stages include: F0 leaves with unrolling pinnae and an indusium of ca. 250 µm (**c**), F1 Nearly fully expanded leaf and an indusium of ca. 800 µm (**d**), and F2 Fully expanded leaves and an indusium of ca. 2 mm. (**e**). Fi = False indusia, S: Sterile stage, F: Fertile stage. **(f–o)** Developmental stages of *E. giganteum*. SAMs producing photosynthetic leaves only in as thin and green at sterile stage S0 (**f**) and thick and brownish-green at sterile stage S1 (**g**) that vary in meristem size (**k**, **l**). Stage F0 has fully differentiated peltate fertile leaves (sporangiophores), that are covered and protected by the sterile leaf sheath and the spore mother cells have not undergone meiosis (**h**, **m**); Stage F1 is characterized by the exposure of half of the strobili above the sterile leaves, of ca. 1 cm and carry mature spores (**i**, **n**). Stage F2 has a fully exposed strobili with sterile leaves only at its base of ca 4 mm and mature spores and expanded elaters (**j**, **o**). Scale bars for c–e = 250 µm; **f–j** = 1 cm; Scale bars **k–o** = 2 mm. Scale bars in magnifications = 50 µm. Black arrowheads indicate the SAM, black arrowhead in insets of **n**, **o** indicate the elaters. Sl = Sterile leaves, Pl = Peltate spore bearing leaves, Sti = sporogenous tissue, S = Spores

To hypothesize the roles of *LEAFY* genes during the reproductive transition in ferns, in addition to those linked to gametophyte-to-sporophyte transitions already reported [39], we evaluated the expression of all *LFY* copies in the ferns *Adiantum raddianum* and *Equisetum giganteum*. These two genes represent extremes in the leaf dimorphism gradient, as *A. raddianum* is monomorphic (i.e., there are no gross morphological differences in sterile and fertile leaves) and *E. giganteum* is holodimorphic (i.e., sterile and fertile leaves vary dramatically in size, shape and position (Fig. 4).

In *A. raddianum* sterile leaves can be separated into fiddleheads (Fd) and young sterile leaves (S), lacking any sign of indusia (i.e., the membranous covering tissue protecting the sori) or sori (i.e., the clusters of sporangia) (Fig. 4a, b). In addition, three fertile developmental stages were established, namely, Stage Fertile F0, F1 and F2, which considered the degree of leaf expansion and the indusia size (Fig. 4c–e). F0 is characterized by leaves with expanding pinnae and an indusium width of ca. 250 µm (Fig. 4c). F1 is characterized by almost entirely extended leaves and an indusium width of ca. 800 µm (Fig. 4d). F2 is characterized by fully extended leaves and an indusium width of 2 mm. (Fig. 4e). Sporangia developmental stages were not specifically described for each stage as sporangia development is asynchronous, meaning that different sporangia are in different developmental stages in a single sori in any given time (Fig. 4a–e).

In *E. giganteum*, we identified two different developmental stages for sterile stems (Fig. 4f, g) and three of reproductive meristems (Fig. 4h–j). Sterile stages correspond to shoot apical meristems (SAMs) producing leaves only (Fig. 4k, l). These were found in two forms, thin and green (Fig. 4f) or thick and brownish-green (Fig. 4g). Although, anatomical sections revealed differences in meristem width, the two forms exclusively produce identical leaves (Fig. 4k, l). The identification of developmental stages in the fertile stems relied on changes of strobilus size, as well as sporangia and spore/elater development (Fig. 4h–j, m–o). Stage Fertile 0 (F0) is characterized by fully differentiated peltate leaves (i.e., sporangiophores), but still covered and protected by the sterile leaf sheath (Fig. 4h, m). In F0, the spore mother cells have not undergone meiosis (Fig. 4m). The Fertile 1 (F1) stage is characterized by the exposure of half of the strobilus above the leaf sheath and by mature spores (Fig. 4i, n). The Fertile 2 (F2) stage is characterized by fully exposed strobilus with leaves only at their base and by mature spores (Fig. 4j, o). In both stages, F1 and F2 elaters (i.e., sterile elongated cells that uncoil in response to changes in humidity, assisting in the dispersal of the spores) can be easily identified (Fig. 4n, o).

**Fig. 5:**
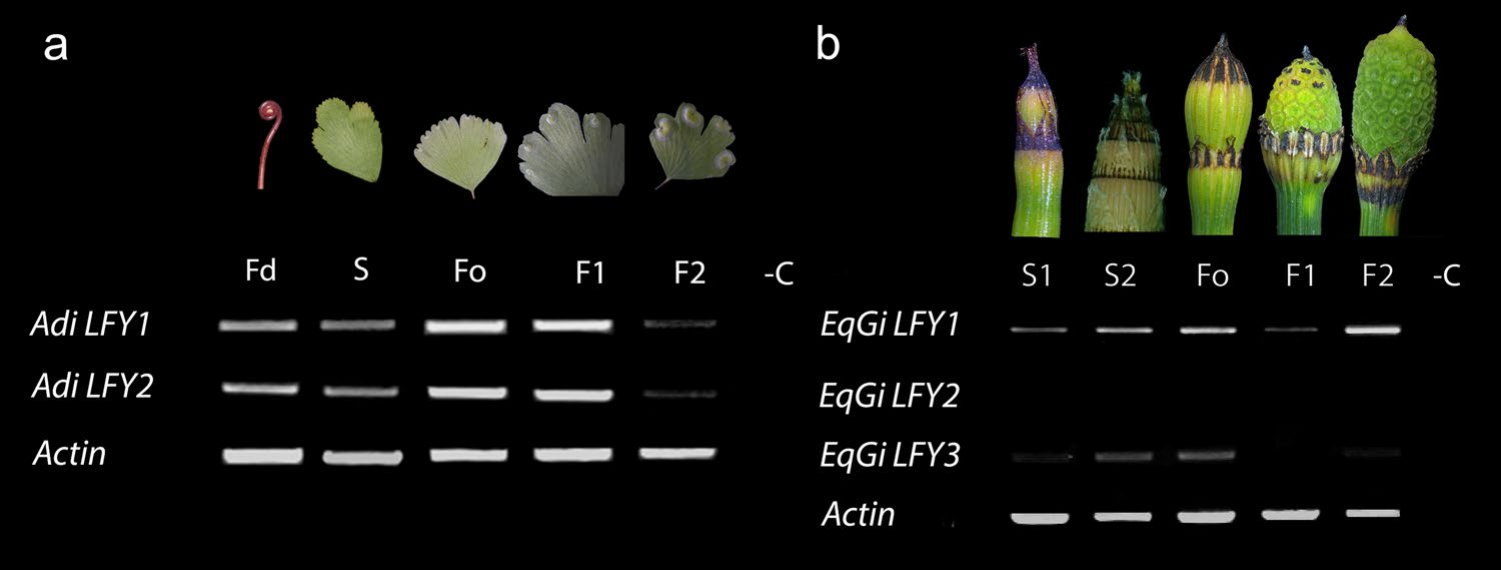
Semi-quantitative expression of *LFY* genes by RT-PCR in selected fern species. **a** Expression of the monomorphic *Adiantum raddianum* (Pteridaceae) paralogs **b** Expression of the holodimorphic *E. giganteum* (Equisetaceae) copies. Fd: Fiddlehead, S: Sterile stage, F: Fertile stage. -C: negative control. *Actin* was use as the positive control

Expression of *LFY* homologs was evaluated in all stages described above. We found two *LFY* copies in the monomorphic *A. raddianum* (Fig. 1), both genes have identical expression patterns with higher expression in fertile stages when compared to fiddleheads and sterile leaves (Fig. 5a; Additional file 7: Fig S4) with a lower level of expression at the F2 stage. On the other hand, in the holodimorphic *E. giganteum* we found three *LFY* paralogs with different expression patterns during reproductive transition. *EqgiLFY2* was undetected in the stages and tissues studied. *EqgiLFY1* is more highly expressed in fertile than sterile stages with two peaks of expression, one at F0 and the second at F2 (Fig. 5b). Finally, *EqgiLFY3* is detected in sterile and until early fertile stages at F0, with very low levels of expression detected in the two latter fertile (F1 and F2) stages.

### In situ hybridization expression of *LFY* homologs in the lycophyte *Selaginella moellendorffii*

**Fig. 6:**
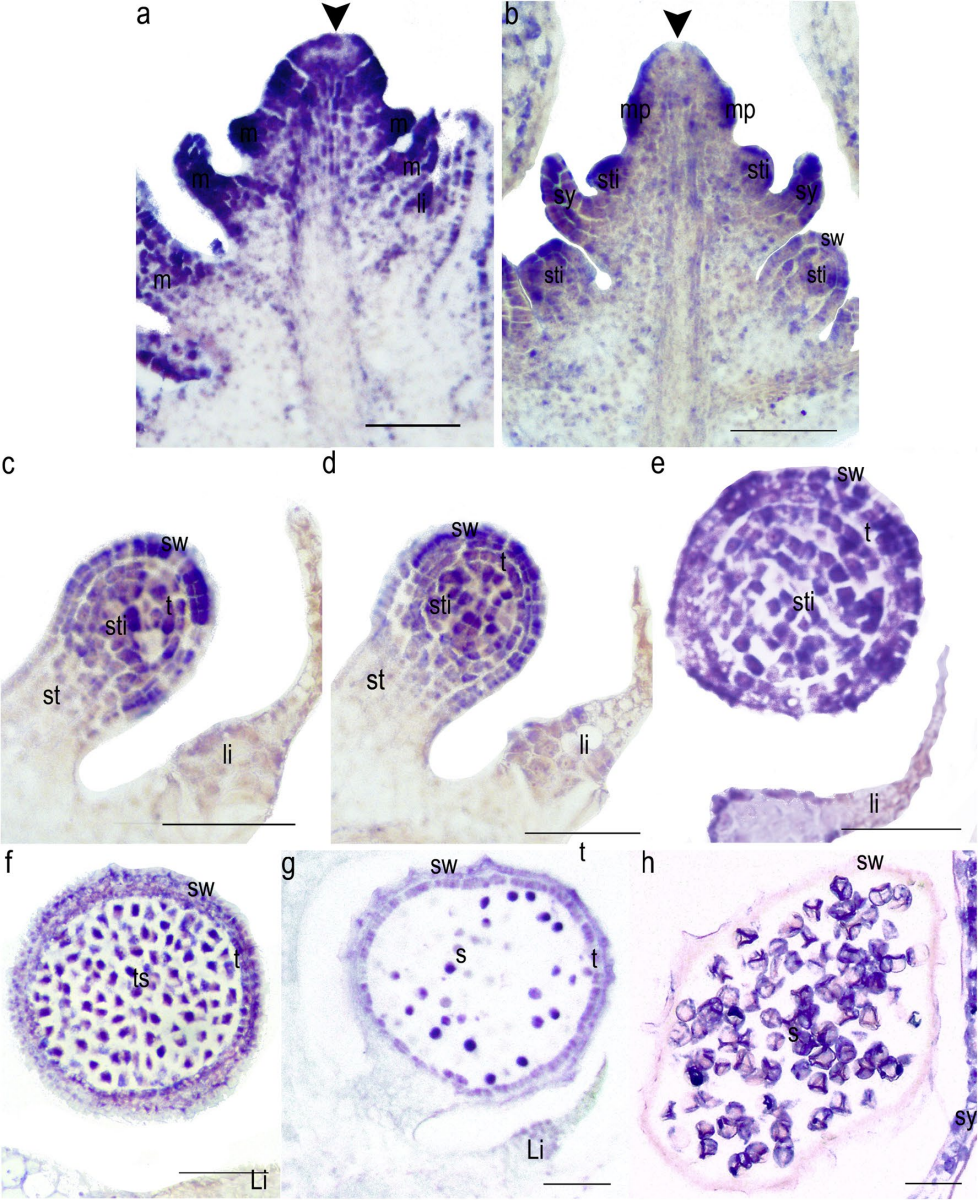
Expression of *Selaginella moellendorffii LFY* genes by in situ hybridization. **a** Vegetative shoot apical meristem (SAM). **b** Reproductive SAM with arising sporophylls and sporangia primordia. **c**–**d** Consecutive developing stages of the sporangium with a developing stalk and a forming sporangial wall surrounding the sporogenous tissue. **e** Two sporangial wall layers enclosing the sporogenous tissue prior to meiosis. **f** Two sporangial wall layers and tapetum. **g** Sporangium after meiosis **h** Mature sporangium with collapsed wall layers and tapetum and fully developed spores. li = ligule, m = microphyll, sti = sporangia, sw = sporangial wall, sy = sporophyll, t = tapetum, ts = tetrads, s = spores. Black arrowheads indicate the SAM. Scale bars = 100 µm

To determine the detailed spatio-temporal expression patterns of the only *LFY* gene identified in the heterosporous lycophyte *S. moellendorffii*, we performed in situ hybridization in vegetative and reproductive shoots following developmental stages identified by Ambrose et al. [44]. *SeMoLFY* is expressed in sterile shoot apical meristems (Fig. 6a), and in reproductive shoots (Fig. 6b). *SeMoLFY* expression within the strobili is detected in the flanks of the meristem, microphyll primordia, sporangia primordia as well as in sporangial tissue and young microphylls (Fig. 6b). When the sporangium wall and stalk are differentiated, *SeMoLFY* expression is broad in all the cells but later in development it starts to be preferentially expressed in the sporangial wall and the sporogenous tissue and turned off in the stalk (Fig. 5c, d). During sporangia maturation, when the tapetum is differentiated, the expression of *SeMoLFY* can be detected in the tapetum itself and in the spores before meiosis (Fig. 6e–-f). During the formation of tetrads, expression of *SeMoLFY* becomes restricted to the spores and the sporangial wall (Fig. 6g), and finally expression is only detected in the spores (Fig. 6h).

## Discussion

Unlike several other transcription factor families important for plant development, the *LFY* gene lineage is usually retained primarily as a single copy in streptophytes and across land plants [5]. A single large scale duplication has been identified in this gene lineage linked to the diversification of seed plants resulting in the *NDLY* and *LFY* paralogs, followed by the loss of *NDLY* in angiosperms while retained in gymnosperms [35, 38, 45]. In this scenario, all other land plants retained single copy pre-duplication *LFY* genes with few species specific duplication events [5, 7, 39, 40, 46]. Interestingly, despite this unusual strong selection acting upon *LFY* single copy genes, their roles are divergent in different plant lineages [5, 40]. Functional data points to plesiomorphic roles of *LFY* genes in the control of cell divisions in the zygote to form the sporophyte in bryophytes [42]. Added evolutionary roles include the maintenance of indeterminate cell fate in both the gametophyte and the sporophyte in ferns [39]. New key functions in reproductive transition are part of the functional repertoire of *LFY* homologs in seed plants. Namely, both *LFY* and *NDLY* are recruited in male and female cone development in gymnosperms [35–38, 45] and *LFY* homologs control floral meristem identity in angiosperms [1, 47]. Finally, *LFY* homologs have also been recruited in maintaining the indeterminate lateral inflorescence meristems in monocots and in forming compound leaves in several legumes [27, 28, 33]. These data challenge the notion of gene duplication as a major driver of gene functional diversification. Instead, it points to changes in protein sequences, specifically in motifs responsible for DNA binding specificity, as key features in the evolution of new functions [5]. To date, the changes in the interaction capabilities of LFY proteins in different plant lineages have been directly linked to sequence changes of *LFY* genes [5, 40].

Contrary to this idea, recent comprehensive phylogenetic analyses have identified *LFY* duplications predating land plant diversification, or at least mosses and liverworts, resulting in ancient duplicates that were lost in tracheophytes [6, 48]. The hypothesis of the existence of ancient duplicates predating land plant diversification is supported with the finding of a few moss *LFY* genes with type I motifs and a few liverwort homologs with type II motifs [6]. Less conflicting arguments have been posed around the evolution of *LFY* genes in tracheophytes, as most phylogenetic analyses to date recover single copy genes with type I motifs in lycophytes and ferns and the already mentioned *NDLY/LFY* duplication in seed plants with the retention of only *LFY* copies in angiosperms. Our analysis does not recover the ancient type I and type II duplications in embryophytes, perhaps because an extensive sampling in bryophytes was not targeted in our study. We were also unable to retrieve the previous reported two *Osmunda* sequences claimed to have promiscuous DNA binding types [6]. In our analysis, we were only able to identify DNA binding type I motifs in lycophyte and fern sequences, the same motif type retained throughout tracheophytes. Our phylogenetic analysis also recovers several local duplications in lycophytes and ferns, some of which had been previously identified [5, 39], and some are new in Isoetaceae in lycophytes and in Equisetales in ferns. Many of the duplications occurring in these lineages could be possibly linked to reported whole genome duplication events [49]. However, most of the duplications found correspond to species-specific paralogs and may be the result of other mechanisms such as tandem repeat replication or retrotransposition as copy numbers can be very different in closely related species.

Understanding the evolution and the putative roles of *LFY* genes in lycophytes and ferns is important as the data will help to clarify the roles of *LFY* homologs present in tracheophytes that are absent in non-vascular plants, and the plesiomorphic functions present in tracheophytes prior to the emergence of *NDLY* and *LFY* paralogs in seed plants. The most comprehensive analysis to date of fern *LFY* genes is that of Plackett et al. [39], which identified two recent *LFY* paralogs in *Ceratopteris richardii* that act partially redundantly in maintaining the indeterminacy of the shoot apex, both in the gametophyte and the sporophyte. However, this same study showed that expression of at least one *LFY* paralog, *CrLFY1* in *C. richardii* expands beyond the shoot apices, specifically to newly emerged leaves as well as in young fertile leaves. This coincides with previous reports identifying higher expression of *C. richardii LFY* genes in shoot tips and circinate reproductive leaves [7]. Similarly, the active expression of *LFY* paralogs in the lycophyte *Isoetes sinensis* in microsporangia and megasporangia points to putative roles in sporogenesis [41], and what has been broadly considered as the reproductive tissue formation by the formation of specialized sporangia with spore mother cells undergoing meiosis. Our data here suggest that several *LFY* homologs in lycophytes (*S. moellendorffii*) and ferns (*Adiantum raddianum* and *Equisetum giganteum*) are highly, and preferentially expressed in fertile stages and the expression is found to be correlated with reproductive tissues (Figs. 5 and 6). If taken together, the expression data available, including that presented here, raises the possibility that *LFY* genes play roles in the formation of sporangia and, if so, in the specialization of reproductive leaf derived tissues. If so, this function is not new or exclusive to seed plant *LFY* homologs but was already present in the ancestor of vascular plants, because it is also present in lycophytes and ferns. Interestingly, expression of the *P. patens PpLFY1* and *PpLFY2* copies was detected in the sporophytes, beyond the early zygote divisions and until the mature sporophyte [42]. A role of *LFY* homologs in sporophyte development was in fact postulated originally in *P. patens* [42], but a role in the induction of the reproductive phase was ruled out based on the lack of complementation of *Arabidopsis lfy* mutants using *PpLFY* genes [40].

There are three major aspects to consider in the assessment of the *LFY* functional capabilities outside of angiosperms:

- 1) Most of the functional data comes from complementation analyses of *Arabidopsis lfy* mutants [37, 40, 41, 45, 50]). The observations showed that sequences closely related to *Arabidopsis* frequently recovered the wild type phenotype, and that, the rescue of *lfy* mutants by gymnosperm, fern and bryophyte sequences decreased in efficiency. Although informative, complementation experiments only allow us to address similarities of the functional capabilities of these genes when compared to the *Arabidopsis* canonical *LFY* gene*.* Therefore, these experiments are very limited to reach conclusions regarding the genes endogenous roles.
- 2) Fern *LFY* genes have pleiotropic early roles in the gametophyte and early sporophyte development, preventing the observation and analyses of their functions during the reproductive transition. The comparative expression data available, does not rule out the possibility that *LFY* genes play roles in the reproductive transition or at least in sporangia formation in lycophytes and ferns, like those recorded in seed plants. If so, an early acquisition of reproductive roles for *LFY* homologs could be traced back at least to the common ancestor of tracheophytes. Also unclear is the putative contribution of species-specific paralogs to gene functional diversification. Our data and that of Yang et al. [41] and Plackett et al. [39] points to some overlap between copies resulting in redundancy but also recover important expression differences between paralogs suggesting some specialization among paralogs. Only the evaluation of the endogenous gene function in ferns and lycophytes, will allow an examination of the contribution of local duplicates to reproductive transition and in turn, assess the functional evolution of the *LFY* gene lineage, one of the core developmental genes in the evolution of land plants.
- 3) DNA binding capabilities have been exclusively tested with flowering plant partners, such as *AG* and *AP1* [9, 40, 51]. Most of the key domains of LFY for floral function have been determined. LFY proteins in gymnosperms, ferns, lycophytes or bryophytes have conserved and easy to identify, oligomerization and DNA binding domains, but exhibit some non-neutral changes, sometimes affecting key positions [6, 41]. For instance, the *C. richardii* LFY proteins changes in amino acid positions within the N-terminal and C-terminal domains have proven to be critical for the recovery of *lfy* mutants in *Arabidopsis* [40].

Our alignment, as well as those of Yang et al. [41] and Gao et al. [6], show that these changes are common across lycophytes and ferns. In parallel, we have also identified motifs 9 in the DNA binding domain and motif 10 in the SAM domain as fern-specific. In addition, studies on the putative protein–protein interactions and DNA-binding properties outside angiosperms are lacking and in turn, LFY partners in lycophytes and ferns remain unknown making it difficult to assess if protein shifts have changed dimerization and/or DNA binding processes endogenously. One critical aspect here is that several genes that are turned on upstream of LFY, such as FLOWERING LOCUS T/TERMINAL FLOWER 1 (FT/TFL1) and MADS-box proteins acting as flowering integrators have lycophyte and fern homologs (Article under review and [44]). For example, the type II classic MADS-box genes from *Selaginella moellendorffii*, *SmMADS1* and *SmMADS6*, are expressed from the earliest stages of sporangia development to mature spores and, therefore, overlap with *SmLFY* expression patterns [44]. More importantly, these MADS-box transcription factors seem to be expressed in an overlapping manner to *LFY* in the sporophytes [52–56]. Future studies could aim at assessing spatial temporal expression of MADS-box genes as well as assessing protein partners in lycophytes and ferns to assess what interactions were in place during sporophyte growth and are not exclusive to angiosperms, similar to evaluations already done in gymnosperms [51, 57]. Understanding the rewiring of genetic regulatory networks for these critical developmental control genes will allow an understanding of the apparent neofunctionalization of *LFY* that emerges in different plant lineages.

## Conclusions

Large-scale and species-specific *LFY* duplications have been identified in lycophytes and ferns. Some of these may be the result of ancient WGD as is the case for Equisetales, while the remaining species-specific copies are probably a result of recent polyploidy or hybridization events. We recovered little protein variation among LFY proteins across land plants. The type I motif that is shared by all tracheophytes is recovered in the DNA binding domain of ferns and lycophytes as expected; but the new motifs 9 and 10 are found to be fern-specific and located in LFY_SAM and the DNA Binding domain, respectively. As both domains are key for DNA binding, further analyses are needed to elucidate downstream factors, and partner proteins that interact during sporophyte growth in lycophytes and ferns. *LFY* genes in the lycophyte *S. moellendorffii* and the two fern species *A. raddianum* and *E. giganteum* show a wide expression among tissues. However, these expression patterns were found to be relatively higher in differentiating young fertile stages and early sporangia developmental stages that then decreases once mature stages are reached. In addition, functional diversification among paralogs is found for *E. giganteum* that matches with that reported for *C. richardii LFY* copies. Roles in reproduction have been reported in gymnosperms and angiosperms, but as the assessment of reproductive function across *LFY* genes have been based on the ability of homologs to complement *Arabidopsis lfy* mutants, it has not been possible to clarify the role of *LFY* outside seed plants. Therefore, the evaluation of endogenous gene function is key to know the contribution of these genes and their duplicates in reproductive transition. Our results allow us to propose a putative role for lycophyte and fern *LFY* homologs in reproductive tissue diffenretiation, indicating that this function may be present in the *LFY* gene lineage prior to the diversification of seed plants. Even though further investigations are needed, our report represents a step forward in the assessment of *LFY* genes in a reproductive context outside seed plants.

## Methods

### Transcriptome generation

Four species representing leaf monomorphism (*Adiantum raddianum*), hemidimorphism (*Anemia villosa*) and holodimorphism (*Equisetum bogotense* and *E. giganteum*) were chosen based on material availability of growing populations close to Medellin (Colombia). The species vouchers were deposited in the herbarium of University of Antioquia (HUA) (C. Rodríguez-Pelayo 1–5). The material was collected in the field and was flash frozen in liquid nitrogen. For *A. raddianum* and *A. villosa* the plant parts collected included fiddleheads as well as fertile and sterile pinnae at different developmental stages. For *E. giganteum* and *E. bogotense* fertile and sterile shoot apical portions at different developmental stages were collected. A detailed morphological description of the stages included for each species can be found below in the RT-PCR expression section.

The tissue was ground using liquid nitrogen and total RNA extraction was carried out using PureLink Plant RNA Reagent (Invitrogen, USA). The total RNA was quantified using a Nanodrop (Thermo, USA) and visually inspected in a 1% agarose gel stained with ethidium bromide. Quality was assessed based on the presence and integrity of ribosomal RNA bands. RNA extractions with concentrations over 200 ng/ul were sent to the sequencing facility (Macrogen, South Korea). RNA-seq experiments were conducted using a TruSeq mRNA library construction kit (Illumina) and sequenced in a HiSeq2000 instrument producing 100 base paired-end reads. The transcriptomes were assembled de *novo* with Trinity V2 at the Centro Nacional de Secuenciación Genómica (CNSG), following default settings [58]. Read cleaning was performed with prinseqlite v0.20.4 with a quality threshold of Q35 [59]. Contig metrics for each species can be find on Additional file 3: Table S1.

### Gene phylogenies

To isolate *LEAFY* genes, sequences from *Arabidopsis thaliana*, *Selaginella moellendorffii*, *Physcomitrium patens* and *Ceratopteris richardii LFY* homologs were used as queries in BLASTN searches in all available ferns and lycophyte transcriptomes and genomes. BLAST searches were made in Phytozome (http://www.phytozome.net/), NCBI, (https://blast.ncbi.nlm.nih.gov/Blast.cgi), and Fernbase (https://www.fernbase.org/), as well as in transcriptome database OneKP (http://www.bioinfodata.org/Blast4OneKP/). Most hits were retrieved as complete coding sequence (CDS) with some partial CDS derived from transcriptomic data. Partial sequences were included only if they had 50% of the ca. 420 amino acids reported for the gene [1], and at least one of the two LFY domains [40]. Using the same strategy, we isolated sequences from our own generated transcriptomes from *Adiantum raddianum*, *Anemia villosa*, *Equisetum giganteum* and *E. bogotense*. These sequences were deposited in GenBank and can be found under the following accession numbers (MW219613, MW219614, MW219618–MW219620, MW219623–MW219625, MW820861–MW820863).

A comprehensive *LEAFY* matrix was built primarily using that of Sayou et al. [5] but integrating all new homologs identified here. All sequences were compiled in Bioedit (http://www.mbio.ncsu.edu/bioedit/bioedit.html) and manually edited to exclusively keep the CDS for all transcripts. Nucleotide sequences were subsequently aligned using the TranslatorX online platform (http://translatorx.co.uk/) implementing the online MAFFT alignment algorithm [60] with default settings. The alignment was refined manually considering the SAM_LFY and DNA Binding domains reported as conserved in *LFY* genes and can be found in Additional file 4: Fig. S3. Maximum likelihood (ML) phylogenetic analyses using the nucleotide sequences were performed in RaxML-HPC2 BlackBox [61] through the CIPRES Science Gateway [62]. Bootstrapping parameters were set for 1000 Bootstrap (BS) iterations. The tree was rooted with Algae *LFY* genes. Trees were observed and edited using FigTree v1.4.4 [63]. All the retrieved fern and lycophytes sequences can be found in Additional file 5: Table S2.

### Identification of protein domains and motifs

To detect both reported and new conserved motifs in LEAFY protein sequences across land plants we selected a total of 34 sequences representing all subclades within the gene lineage. Sequences were permanently translated and uploaded as amino acids to the online Multiple Em for Motif Elicitation (MEME) server (http://meme-suite.org/) and run to find up to 10 motifs with the default options [64]. The motifs retrieved by MEME are reported according to their statistical significance. The MEME suite finds, in the given sequences, the most statistically significant (low *E* value) motifs first. The *E* value of a motif is based on its log likelihood ratio, width, sites, and the size of the set. We numbered the motifs following the statistical significance resulting in the analyses.

### Expression analyses by Reverse Transcriptase PCR (RT-PCR)

To examine and compare the expression patterns of *LFY* genes in monomorphic, hemidimorphic and holodimorphic ferns we dissected different plant parts and targeted a range of developmental stages for *Adiantum raddianum*, and *Equisetum giganteum*, as described in the results section of RT-PCR expression.

We dissected and flash froze meristems, fiddleheads, and fertile and sterile pinnae at the developmental stages described previously for the two ferns species. Total RNA was prepared from dissected tissues using the same protocol described above. RNA samples were treated with DNAseI (Roche, Basel, Switzerland) to remove DNA contamination and later quantified with a NanoDrop 2000 (Thermo Scientific, Waltham, MA, USA). Three micrograms of RNA were used as a template for cDNA synthesis (SuperScript III RT, Invitrogen) using OligodT primers. The cDNA was used undiluted for amplification reactions by RT-PCR. To ensure specificity for amplification of each copy, the primers were designed in the regions outside of the conserved domains (Additional file 6: Table S3). Each amplification reaction incorporated 9 μL of EconoTaq (Lucigen, Middleton, WI, USA), 6 μL of nuclease-free water, 1 μL of BSA (bovine serum albumin) (5 μg/mL), 1 μL of Q solution (betaine 5 μg/μL), 1 μL of forward primer (10 mm), 1 μL of reverse primer (10 mm), and 1 μL of template cDNA, for a total reaction of 20 μL. Thermal cycling profiles followed an initial denaturation step (94 °C for 30 s), an annealing step (50–62 °C for 30 s) and an extension step with polymerase (72 °C for up to 1 min) repeated for 30–40 amplification cycles. As endogenous controls we tested *Actin1 *[65] as a control for all samples. Finally, the PCR products were run on a 1.0% agarose gel stained with ethidium bromide and digitally photographed using a Whatman Biometra® BioDoc Analyzer. To provide a more “quantitative” analysis of band brightness we converted our raw image data using imageJ. We counted the number of pixels and compared it to the ACTIN band. Visualization of replicates is found in Additional file 7: Fig. S4.

### Developmental series of *Equisetum giganteum*

Sterile and reproductive shoots were collected in the field and immediately fixed in formaldehyde–acetic acid–ethanol (FAA; 3.7% formaldehyde: 5% glacial acetic acid: 50% ethanol). For light microscopy, fixed material was manually dehydrated through an alcohol–histochoice series and embedded in Paraplast X-tra (Fisher Healthcare, Houston, Texas, USA). The samples were sectioned at 10–20 μm with an RM2125 RTS (USA) rotary microtome. Sections were stained with Johansen’s safranin and 0.5% Astra Blue [66] and mounted in Permount (Fisher Scientific, Pittsburgh, Pennsylvania, USA). Sections were viewed and digitally photographed with a Zeiss Axioplan compound microscope and Zeiss stereo microscope equipped with an AxioCamERc5s digital camera with ZEN software.

### In situ hybridization

In situ hybridization experiments were performed for the *Selaginella moellendorffii LFY* homolog. Thus, we used meristematic vegetative portions and strobili and followed sporangium development stages established by previous descriptions in other *Selaginella* species [12, 44, 67, 68]. The material was fixed for 3 h under vacuum in freshly prepared FAA (50% ethanol, 3.7% formaldehyde and 5% glacial acetic acid). The material was dehydrated through an alcohol‐histochoice series and embedded in Paraplast X‐tra (Fisher, Waltham, MA, USA) with optimizations made by A. Vasco (i.e., fixation time 3 h; infiltration solution changes every 3 h and hybridization temperature fixed at 55 °C). Samples embedded were maintained at 4 °C until use. The tissue was sectioned at 8–10 μm on a Microm HM3555 rotary microtome.

DNA template for the *S. moellendorffii SeMoLFY* probe was obtained by PCR amplification of a 500 bp fragment. The fragment was cleaned using the QIAquick PCR purification kit (Qiagen, Valencia, CA, USA). Digoxigenin labeled RNA probe was prepared using T7polymerase (Roche, Switzerland), murine RNAse inhibitor (New England Biolabs, Ipswich, MA, USA), and RNA labeling mix (Roche, Switzerland) according to the manufacturer protocols. In situ hybridization following the protocol that has been described previously optimizing incubation temperatures for each probe and tissue [5, 69, 70]. The sections were hybridized overnight at 55 °C. In situ hybridized sections were subsequently dehydrated and permanently mounted in Permount (Fisher, Waltham, MA, USA). All sections were digitally photographed using a Zeiss Axioplan microscope equipped with a Nikon DXM1200C digital camera. Sense probe controls are shown in Additional file 8: Fig. S5).

## Supplementary Information

**Additional file 1: Figure S1.** ML analysis of the LFY gene family. **a**. Summary tree including sequences from algae, bryophytes and tracheophytes. **b**. ML analysis of the LFY family including algae and land plant sequences. Yellow stars indicate large duplication events. Number on each node indicate the bootstrap value (BS). Black arrowheads point to sequences isolated in this study. The colors correspond to the conventions on the bottom left. Scale: 0.3

**Additional file 2: Figure S2.** Protein sequences of the LFY family. For a total of 34 representative land plant sequences belonging to: Arabidopsis thaliana, Ceratopteris richardii, Azolla filiculoides, Salvinia cucullata, Equisetum giganteum, E. bogotense, Adiantum raddianum, Anemia villosa, Selaginella moellendorffii, Physcomitrium patens and several algae species. The two characteristic domains of LFY proteins reported by Sayou et al. [5, 9] are boxed. Blue arrowheads point to the key positions for DNA binding reported by Sayou et al. [5] .Black arrowheads point to the conserved section among fern sequences.

**Additional file 3: Table S1.** Contig metrics.

**Additional file 4: Figure S3.** Complete alignment in fasta for LFY homologs.

**Additional file 5: Table S2.** LFY sequences complete data set.

**Additional file 6: Table S3.** Primer sequences for fern and lycophyte *LFY* genes used here for RT-PCR and ISH.

**Additional file 7: Figure S4.** Quantification of *LFY* homologs expression using Image J onto gel images with three biological replicates.

**Additional file 8: Figure S5.** Sense experiments for ISH in the meristem (SAM) and in reproductive tissue (See also Zumajo-Cardona et al. [44]).

## Data Availability

Sequences generated in this work were deposited in GenBank and can be found under the following Accession Numbers: MW219613, MW219614, MW219618–MW219620, MW219623–MW219625, MW820861–MW820863.

## Abbreviations

LFY: LEAFY
NDLY: NEEDLY
FLO: FLORICAULA
AGL24: AGAMOUS-LIKE 24
SVP: SHORT VEGETATIVE PHASE
SOC1: SUPRESSOR OF OVEREXPRESSION OF CONSTANS
AP1: APETALA1
SP: SEPALLATA
PI: PISTILLATA
AP3: APETALA3
AG: AGAMOUS
BSA: Bovine serum albumin
Q: Betaine
ML: Maximum likelihood
BS: Bootstrap
MEME: Multiple Em for Motif Elicitation
qRT-PCR: Quantitative polymerase chain reaction
RT-PCR: Reverse transcription polymerase chain reaction
SAM: Shoot apical meristem
WGD: Whole genome duplication
