# Supplementary figures and images for: Evolution and expression of LEAFY genes in ferns and lycophytes
Source: EvoDevo. 2022 Jan 8;13:2. doi: 10.1186/s13227-021-00188-9 (PMC8742948; doi:10.1186/s13227-021-00188-9)

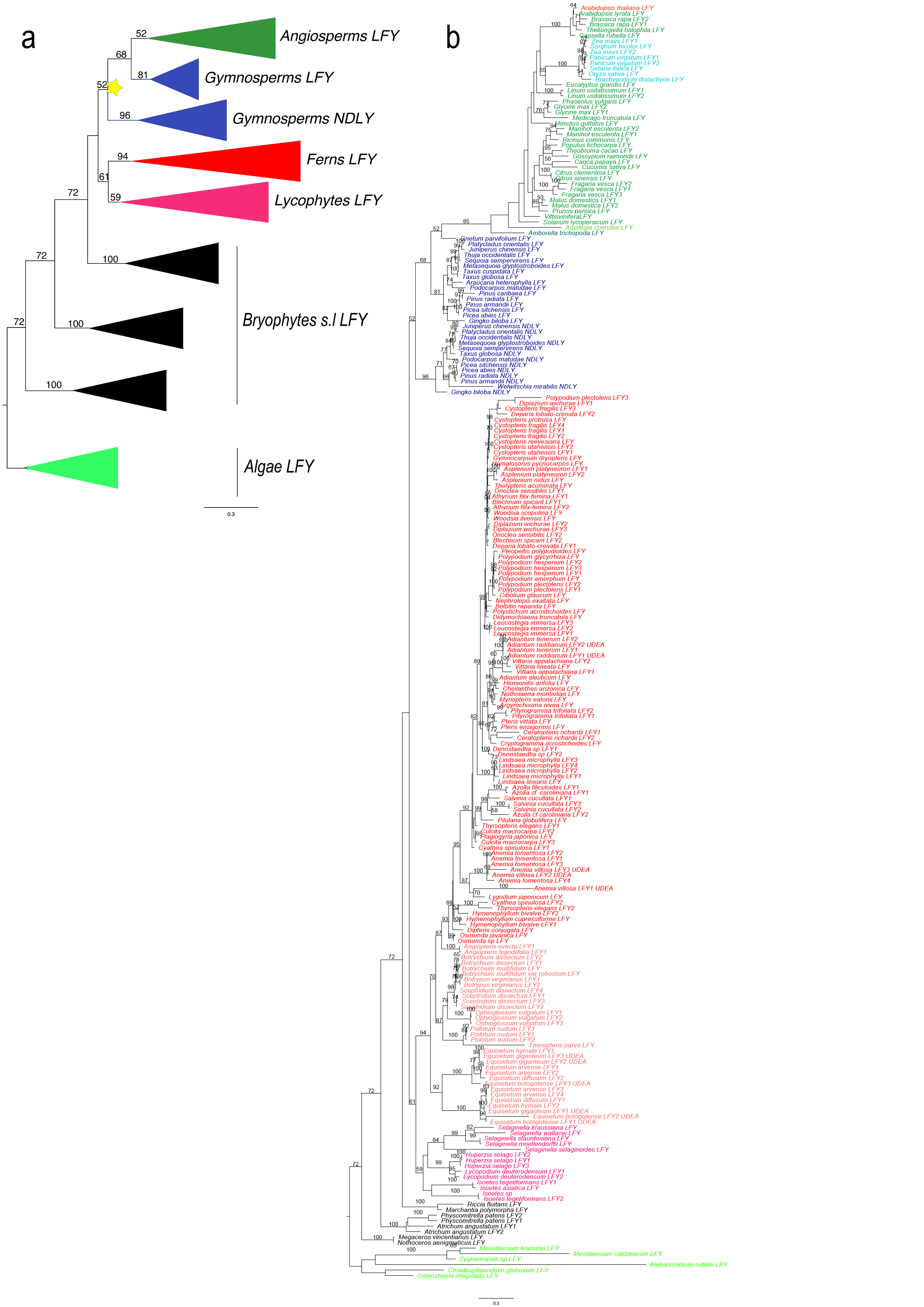

Supplement: Supplementary file 1 — Additional file 1: Figure S1. ML analysis of the LFY gene family. a. Summary tree including sequences from algae, bryophytes and tracheophytes. b. ML analysis of the LFY family including algae and land plant sequences. Yellow stars indicate large duplication events. Number on each node indicate the bootstrap value (BS). Black arrowheads point to sequences isolated in this study. The colors correspond to the conventions on the bottom left. Scale: 0.3 [file 13227_2021_188_MOESM1_ESM.tif]

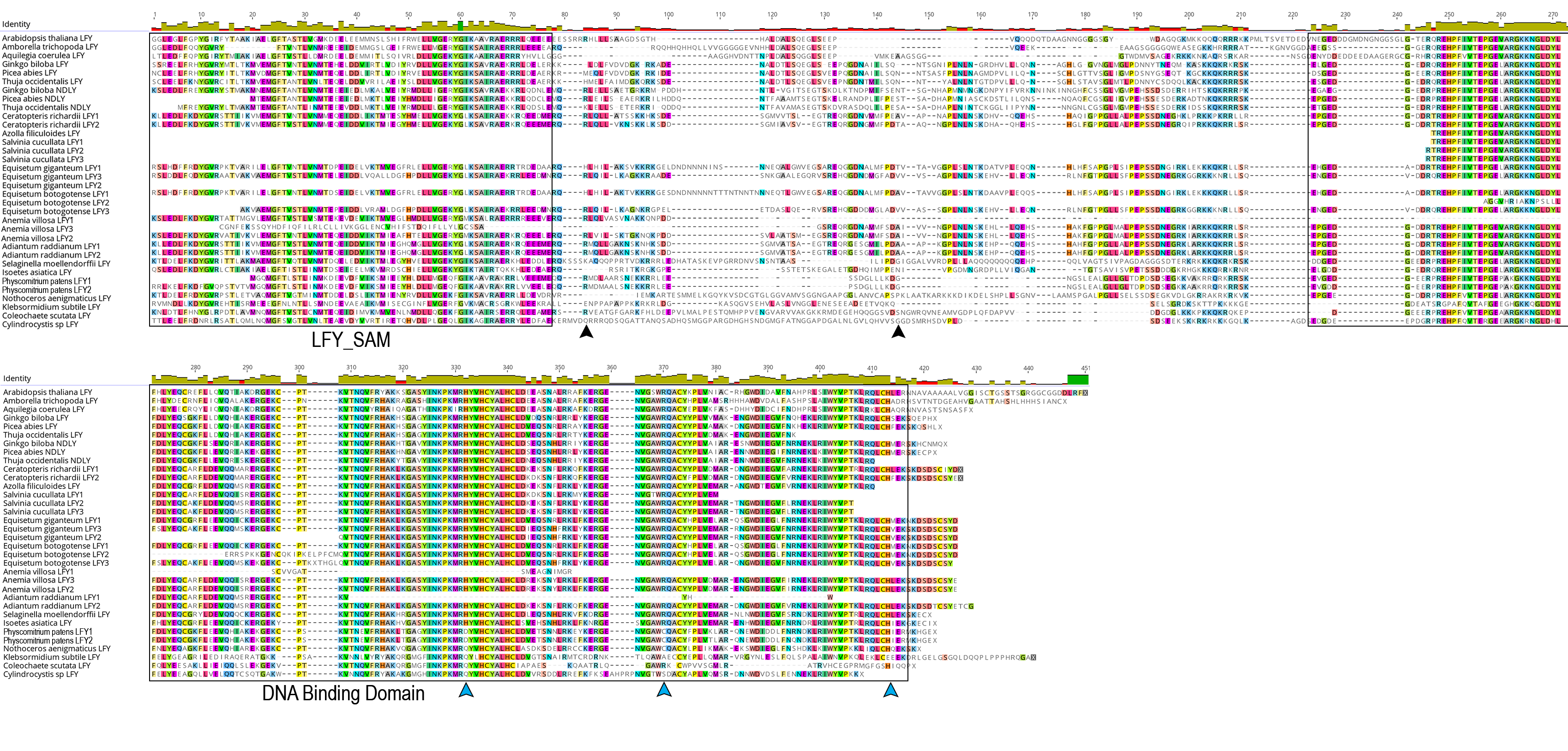

Supplement: Supplementary file 2 — Additional file 2: Figure S2. Protein sequences of the LFY family. For a total of 34 representative land plant sequences belonging to: Arabidopsis thaliana, Ceratopteris richardii, Azolla filiculoides, Salvinia cucullata, Equisetum giganteum, E. bogotense, Adiantum raddianum, Anemia villosa, Selaginella moellendorffii, Physcomitrium patens and several algae species. The two characteristic domains of LFY proteins reported by Sayou et al. [5, 9] are boxed. Blue arrowheads point to the key positions for DNA binding reported by Sayou et al. [5] .Black arrowheads point to the conserved section among fern sequences. [file 13227_2021_188_MOESM2_ESM.tif]

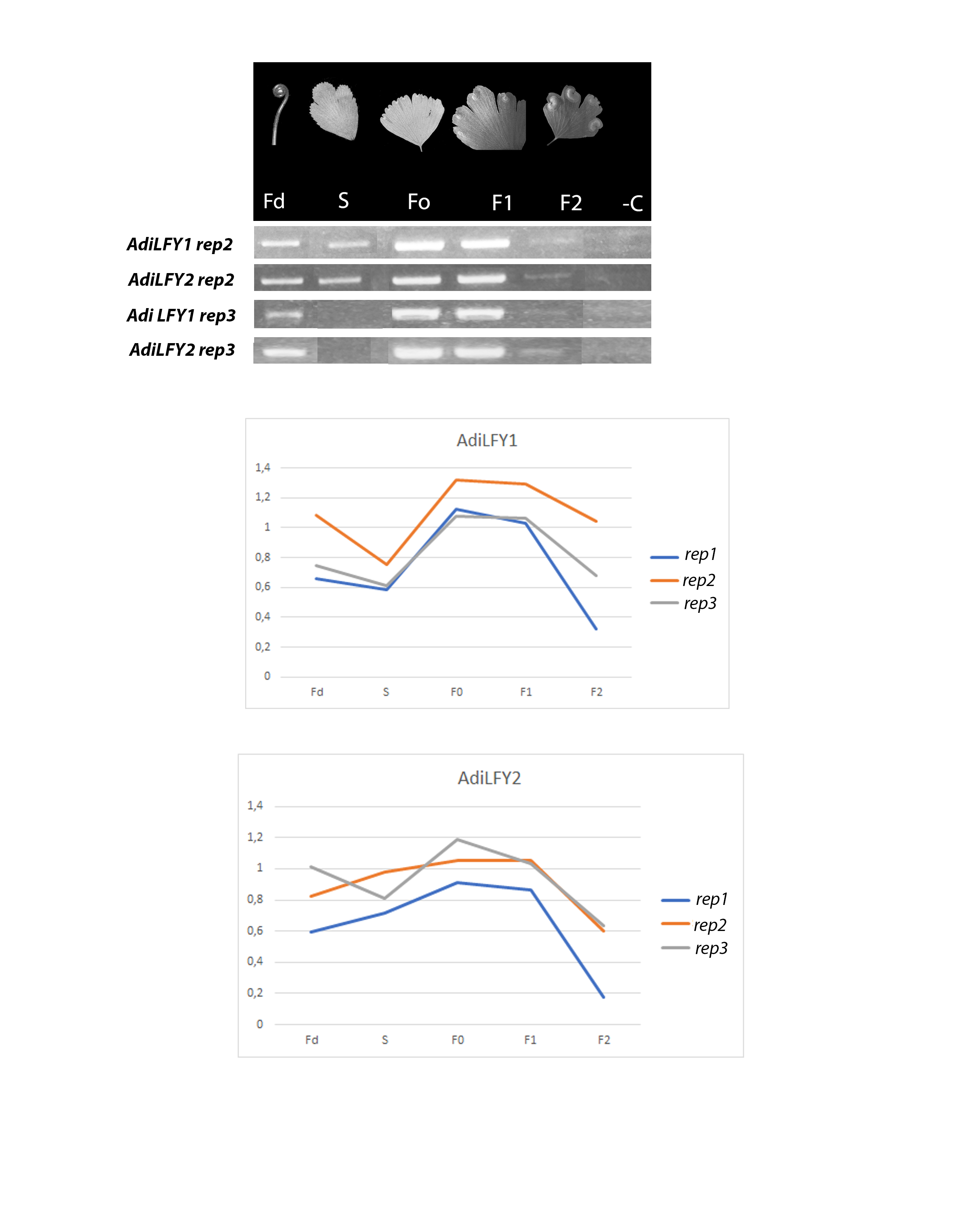

Supplement: Supplementary file 7 — Additional file 7: Figure S4. Quantification of LFY homologs expression using Image J onto gel images with three biological replicates. [file 13227_2021_188_MOESM7_ESM.tif]

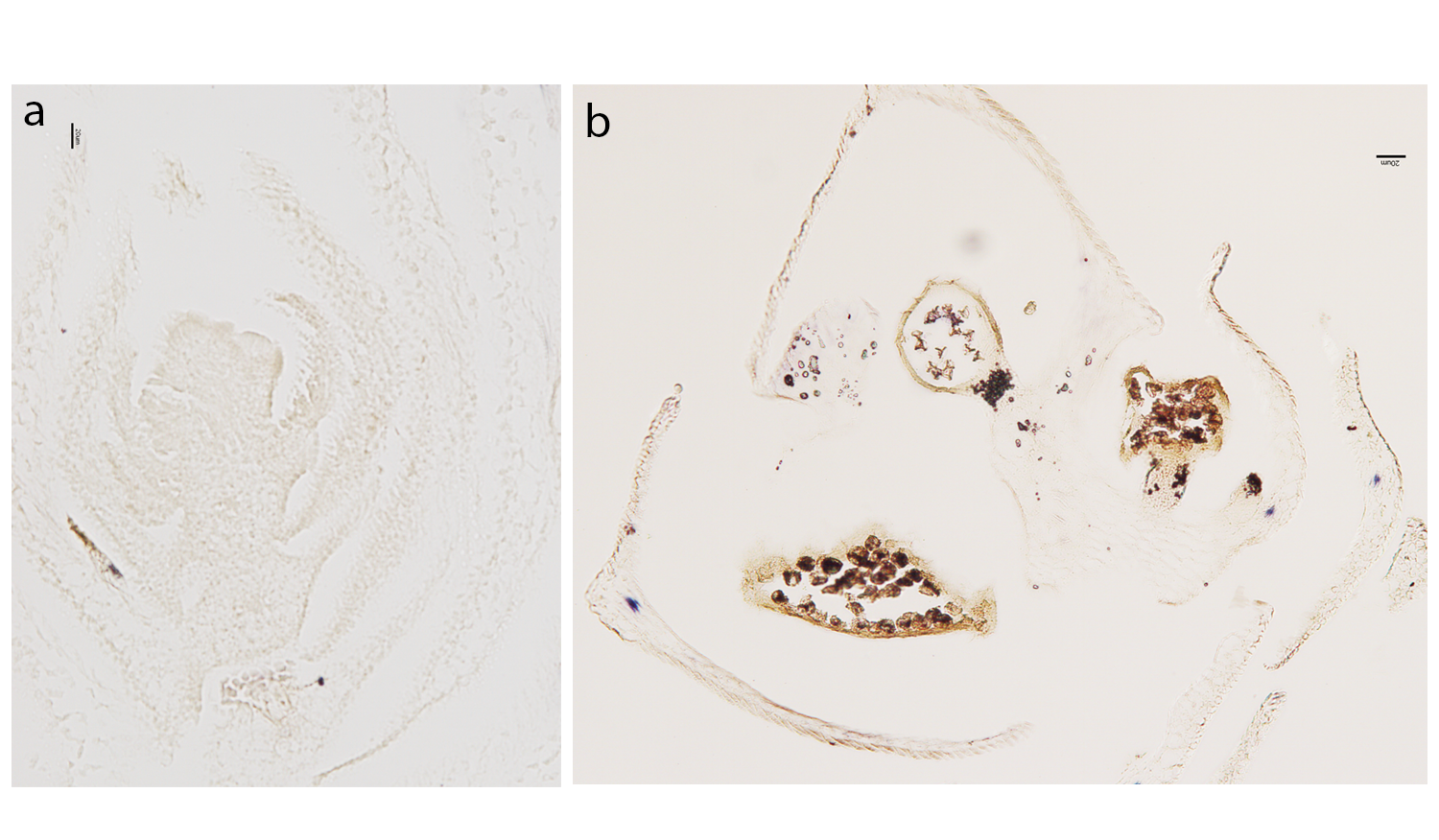

Supplement: Supplementary file 8 — Additional file 8: Figure S5. Sense experiments for ISH in the meristem (SAM) and in reproductive tissue (See also Zumajo-Cardona et al. [44]). [file 13227_2021_188_MOESM8_ESM.tif]
